# New Sensor Data Structuring for Deeper Feature Extraction in Human Activity Recognition †

**DOI:** 10.3390/s21082814

**Published:** 2021-04-16

**Authors:** Tsige Tadesse Alemayoh, Jae Hoon Lee, Shingo Okamoto

**Affiliations:** Department of Mechanical Engineering, Graduate School of Science and Engineering, Ehime University, Matsuyama 790-8577, Japan; tsgtdss583@gmail.com (T.T.A.); okamoto.shingo.mh@ehime-u.ac.jp (S.O.)

**Keywords:** human activity recognition, inertial measurement unit sensors, deep learning, convolutional neural network, input adaptation

## Abstract

For the effective application of thriving human-assistive technologies in healthcare services and human–robot collaborative tasks, computing devices must be aware of human movements. Developing a reliable real-time activity recognition method for the continuous and smooth operation of such smart devices is imperative. To achieve this, light and intelligent methods that use ubiquitous sensors are pivotal. In this study, with the correlation of time series data in mind, a new method of data structuring for deeper feature extraction is introduced herein. The activity data were collected using a smartphone with the help of an exclusively developed iOS application. Data from eight activities were shaped into single and double-channels to extract deep temporal and spatial features of the signals. In addition to the time domain, raw data were represented via the Fourier and wavelet domains. Among the several neural network models used to fit the deep-learning classification of the activities, a convolutional neural network with a double-channeled time-domain input performed well. This method was further evaluated using other public datasets, and better performance was obtained. The practicability of the trained model was finally tested on a computer and a smartphone in real-time, where it demonstrated promising results.

## 1. Introduction

HAR (human activity recognition) is an active research field that focuses on identifying human activities from a visual or sensor input. The growing interest in HAR comes from its necessity in health monitoring [1], human–computer interactive systems, monitoring systems [2,3], among others. The activities can be static or dynamic including walking, sitting, bike riding, jogging, eating, reading, and washing. Data for HAR training are mainly acquired from non-visual sensors such as IMUs (inertial measurement units) and sEMG (surface electromyography) [2,4,5,6,7,8,9,10,11,12,13], visual sensors such as cameras [14,15,16], and a combination of both [17,18]. In addition to its costs and privacy issues, visual-based HAR provokes extra work in image processing including locating subjects in the image frames. On the contrary, owing to the growth of portable smart devices with embedded sensors, IMUs are omnipresent and can be used almost without restrictions. Moreover, wearable devices can be continuously used indoors or outdoors while safeguarding users’ privacy. However, wearable sensors provide relatively limited information compared to cameras.

Owing to their ubiquity and embedded sensor diversity, smartphones have been commonly utilized to develop HAR solutions [5,19,20]. The advantages of smartphones, when compared to other wearable devices, are their capability to capture and process data and their reliability in transmitting and receiving data when connected with other devices. Accelerometers and gyroscopes are the most used sensors for representing human body acceleration and movement direction. Therefore, these sensors have allowed for the extraction of diverse information from the motion of a person that can be used to recognize physical activities [6,8,21,22,23].

HAR can be regarded as a pattern recognition problem. Magnificent progress has been made by conventional pattern recognition methods on HAR through classical machine learning algorithms such as hidden Markov models [24,25,26,27], decision trees [28,29,30], SVMs (support vector machines) [5,29,31,32,33], and naive Bayes [34,35,36]. Even though these methods achieve excellent results when few and lower-dimensional input data exist, certain domain knowledge or a controlled environment is required. This, in turn, results in learning only slight activities [37,38]. Hence, their performance is limited in terms of the classification accuracy and generalization of a model.

According to recent surveys, HAR algorithmic implementation is heading in the direction of deep learning [39]. This is due to the rapid advancement of deep learning methods and their unparalleled performance in many areas such as object recognition, natural language processing, and image processing. Additionally, deep learning-based neural network training has been recently used even in bioinformatics [40,41]. Unlike traditional pattern recognition approaches, deep learning can alleviate the burden of engineering the extraction of features from the input data by learning high-level and meaningful features through neural networks [6,42,43]. It is undisputed that CNNs (convolutional neural networks) and RNNs (recurrent neural networks) have dominated HAR methods over the last couple of years. For its temporal and spatial feature exploitation capability, the CNN has been the champion for time series-related classification problems as well [4,9,10,11,20,38]. On the contrary, for their excellent achievements on sequential data, RNN models are potential neural networks for time series IMU sensor data of a HAR problem [4,44,45,46]. Before the motion data are provided to the deep-learning algorithms, an input adaptation must be performed to influence the training performance of the networks. Hence, these time series motion data were transformed and structured into various forms to obtain better classification results.

In this study, a multi-function iOS application software for smartphones was developed to assist in data handling and real-time recognition. The raw data collected were restructured to increase its dimensionality so that it was easy to exploit the spatial and temporal axis features of the motion data. This was carried out by duplicating the spatial data of the IMU data. This improves the accuracy of the neural network models. The resultant models were made to be as light as possible to reduce the computational cost. The trained model was tested on a computer and a smartphone for real-time motion recognition to check its practicability. This methodology was adopted from a previous conference paper [47].

The main contribution of this study is a better input adaptation method for sensor-based human activity recognition. This was carried out by restructuring raw sensor data in a special manner to improve the performance of neural networks. Duplicated triaxial IMU data were stacked vertically with the intention of extracting spatial features during neural network training. Restructured data were formatted into single and double-channels in an attempt to obtain a better structure in terms of lightness and efficiency. This method is superior to existing state-of-the-art systems.

The remainder of this paper is organized as follows. In the first section, an introduction to HAR and related works are discussed. Section 2 presents the background and related works section. Section 3 and Section 4 mainly focus on input adaptation and neural network architectures developed. After the results are discussed in Section 5, the concluding statements and future work are presented.

## 2. Background and Literature Review

HAR methods intend to understand a person’s activity behaviors to enable computing systems to proactively assist users in various fields [7]. For instance, this can be applied to an elderly person who uses a robotic assistant to walk upright and sound. In this case, the assistive robot can be automatically activated when the person starts to walk and continuously monitors their movement using the wearable sensor’s data.

### 2.1. Conventional Pattern Recognition

Conventional PRs (pattern recognitions) can fall into two major classes: supervised and unsupervised learning. The difference is the availability of labeled output data, where the former has it, but not the latter. Supervised learning attempts to obtain a mathematical model depending on the input–output relation, whereas unsupervised learning identifies patterns from the input data through preprocessing steps which are also required, including feature extraction, vectorization/segmentation, normalization, standardization, and projection [48].

Some of the most common conventional PR algorithms are as follows: (a) naive Bayes: probabilistic classifiers based on the application of Bayes’ theorem to presumably independent features [34,35,36]. (b) Support vector machine: uses a hyperplane to separate data samples into two classes by maximizing the distance between the hyperplane and the data instances on either side [5,13,29,31,32,33]. (c) Random forests: an ensemble learning method that comprises a large number of decision trees for classification or regression [26,49,50]. (d) Decision trees: in the case of decision trees, tree-like decision rules are used to sort out data instances based on the features or data values [28,29,30]. (e) kNN (k-nearest neighbors): stores all available cases and classifies new cases based on resemblance measures [19,51,52,53]. Lastly, (f) neural networks have also been used in previous studies [20,54,55,56].

**Figure 1:**
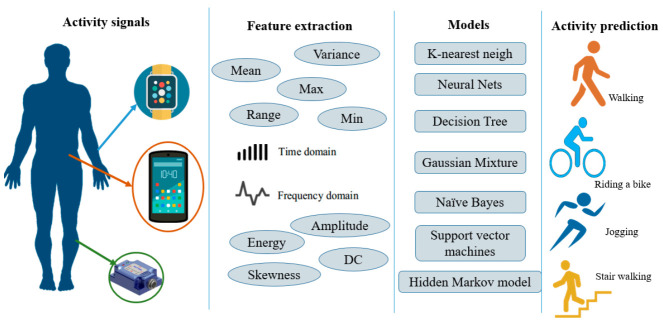
Conventional PR for a sensor-based HAR.

Figure 1 presents a typical flowchart of the HAR using conventional PR approaches. First, raw motion data inputs were collected from various types of sensors (smartphones, watches, among others). Second, statistical features were heuristically extracted from readings based on human expertise [37,38,42], such as the mean, variance, and amplitude in traditional machine learning approaches [57]. Finally, these statistical features serve as inputs to train a conventional PR model for activity inference.

### 2.2. Deep Learning

**Figure 2:**
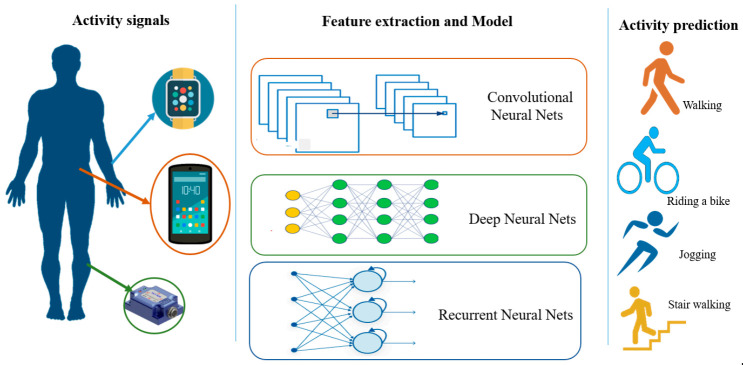
Deep learning for a sensor-based HAR.

Figure 2 shows how deep learning works for HAR with different types of networks. Compared to Figure 1, the feature extraction and model building steps are combined into one step under the deep learning modeling of various neural networks. In this step, automatic feature learning through a network and high-level characteristic extraction by the deep layers is suitable for complex activity recognition tasks.

Nowadays, deep learning is being used in several fields such as supporting medical teams in medical diagnostics [58,59], natural language processing [60,61], agriculture [62,63], obstacle detection, and learning control policies in robotics [64,65,66], and spectral and spatial information processing in remote sensing [67]. It has achieved a promising result in these diverse fields. Moreover, the development of new deep learning algorithms, new devices capable of fast deep learning training, is enabling it to work with diverse input data including text, images, time series, audio. However, it highly relies on the quantity of the dataset, which is difficult to collect and time-consuming. This limits the application range of deep learning.

There are two paradigms related to solving sensor-based HARs using deep learning: data-driven and model-driven. Data-driven approaches treat each input dimension as a channel. For instance, a three-axis accelerometer data is assumed to have three channels: x, y, and z. Subsequently, the learning algorithm tries to learn by determining the distinctions among input data [9]. By using 1D convolution in the temporal window, [38] further improved it by unifying and sharing weights within a multiple-sensor CNN implementation. Such approaches ignore the spatial dependence within a sensor and among multiple sensors when multiple sensors are used.

In model-driven approaches, the input data are reshaped or transformed into a form that can produce a better result. One method is to resize the inputs to a virtual 2D image to adopt a 2D convolution. This method usually pertains to non-trivial input-tuning techniques. In [10], zero pads were introduced into the combined virtual image of the samples to separate different sensor values from each other. In [11], a different complex algorithm was also designed to transform the time series into a larger 2D image. Even though these approaches are better, adding rows of zeros introduces unwanted information, and the network size increases. Similarly, larger 2D virtual images consume resources, making it difficult to implement them in actual devices.

RNNs and CNNs are widely used for training end-to-end networks to extract high-level features. According to a recent survey, the CNN is leading a group of deep-learning methodologies for HAR. However, in terms of the average accuracy of CNN papers recorded over the last five years, it falls slightly behind the RNN methods [39]. This could be improved with a better input adaptation method wherein data instances are prepared with the CNN learning methodology in mind. Among the previous works, ref. [68] used a CNN and LSTM (long short-term memory) for emotion detection over multimodal raw data. Data from different sensors were collected and fused before being assigned to the deep-learning layers. In [69], a 1D CNN was also used to recognize indoor activities using data collected from multiple sensors including accelerometers, gyroscopes, a barometer, and a magnetometer. Another study [70] performed a 2D CNN over the frequency-domain representation of each tri-axial accelerometer and gyroscope, which were later fused to obtain the predicted activity. Other studies such as [4,9,10,11,20,38,71,72] have also employed CNN-based HAR systems.

On the contrary, RNNs are the second most used neural network models for HAR [39], in addition to their excellent speech recognition and natural language processing. In [73,74], a real-time HAR was implemented using LSTM and an SVM, respectively, over raw motion data. In addition, [44,45], are among the works conducted by HAR using a RNN. A ConvLSTM (convolutional-long short-term memory) was introduced in [75] for rainfall forecasting. It is an LSTM with kernels and convolutions rather than element-wise multiplication.

Unsupervised and semi-supervised learning methods such as an ensemble of autoencoders have been introduced recently in HAR recognition [76]. For their specialty in reducing data dimensionality, autoencoders are good for a lower computational cost. A similar method, named random projection, used probabilistic methods to classify subjects and activities by reducing feature dimensions [77].

The restricted Boltzmann machine (RBM) has been also utilized in HAR. RBMs are non-deterministic generative deep learning models with only two types of nodes—hidden and visible nodes connected by a fully bipartite graph. RBMs can be trained in supervised or unsupervised fashions. Varieties of RBM architectures have been used for pre-training and multi-modal sensor data [78,79,80]. To enhance the performance of a deep learning method, some previous studies used a hybrid model where a different neural network layer is added into the existing network as a complementary [46,81].

Almost all previous studies focused on choosing appropriate neural networks and selecting the type and quantity of sensors. Little attention has been given to input data shaping and the real-time verification of developed systems. This paper will positively contribute to addressing the concerns of HAR systems.

## 3. Data Collection and Management Methods

**Figure 3:**
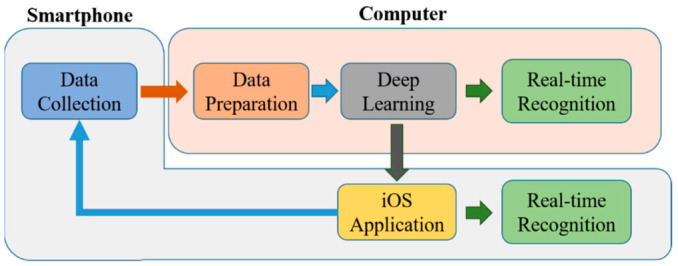
System overview.

The overall process of the methodology used is summarized in the following Figure 3.

### 3.1. Data Collection

Although some HAR approaches can be generalized to various sensor modalities, most of them are specific to certain types. According to [82], sensors lie mainly in three modality categories: body-worn sensors, object sensors, and ambient sensors. For their close contact positioning and capability to capture human kinetics, wearable sensors were chosen herein and will be discussed below.

**Figure 4:**
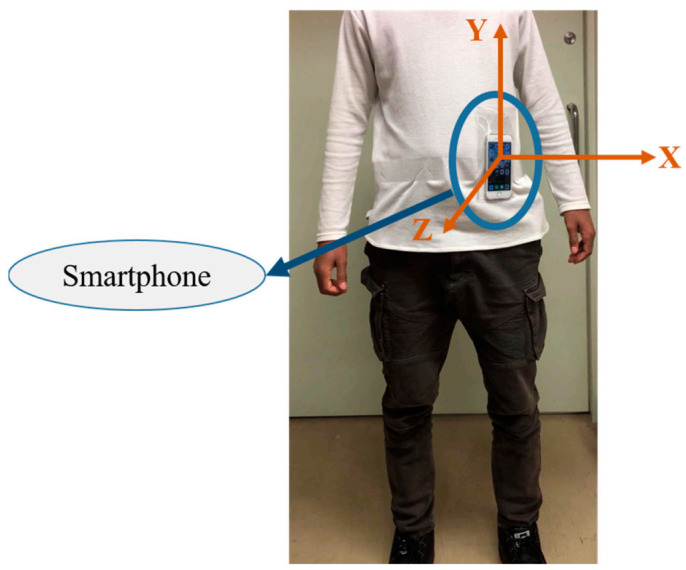
Smartphone (iPhone) attached to the waist as a motion sensor.

Wearable sensors such as accelerometers, magnetometers, and gyroscopes are commonly utilized sensor modalities to capture human movement for HAR. The linear acceleration and angular velocity of a person change according to human body movements. Thus, daily human activities can be inferred from the accelerometer and gyroscope data. Nowadays, these sensors are embedded in several electronic devices that are used daily, such as smartphones, watches, bands, glasses, and helmets. For its omnipresence and reliable communication feature, a smartphone was used as an IMU sensor (only its accelerometer and gyroscope, in this case) attached to the waist of the human body, as shown in Figure 4.

Body-worn sensors are widely used in deep learning-based HARs [9,11,21,38,43]. Among these works, the accelerometer sensor is the one most adopted. However, gyroscope and magnetometer data are frequently used together with accelerometer data in several HAR studies. Predominantly, activities of daily living and sports are mainly recognized by the use of these sensors.

**Figure 5:**
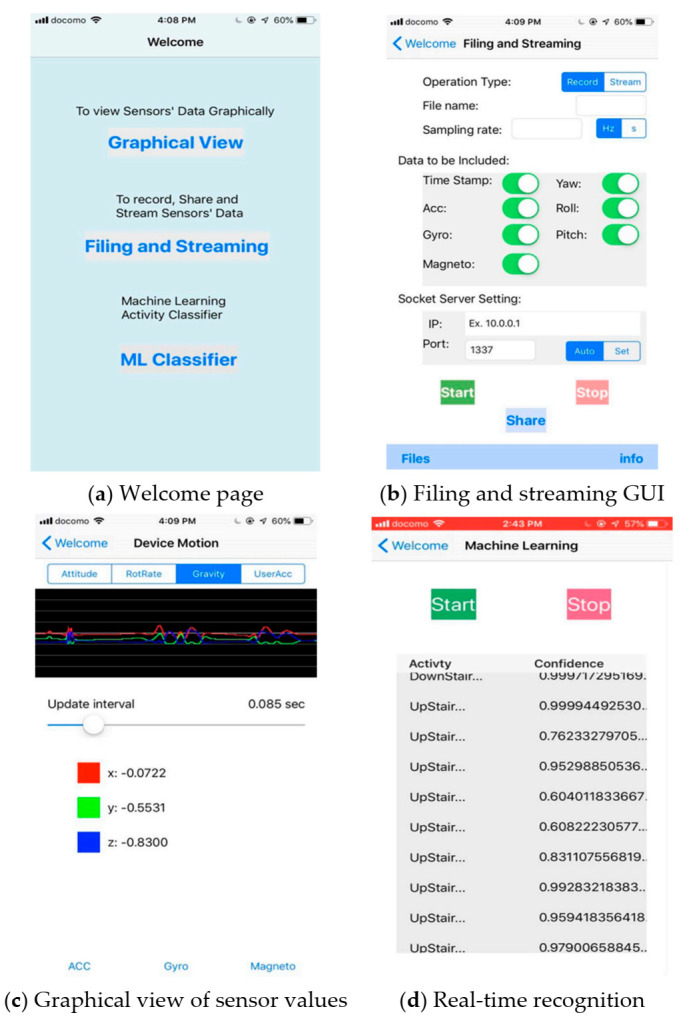
Graphical user interface of the iOS application software developed.

An IMU (inertial measurement unit) sensor embedded in an iPhone 7, providing a 3-axis accelerometer and gyroscope data, was employed as a device for measuring the human motion kinematics. To handle sensor data in a simplified way, an exclusive iOS application software, shown in Figure 5, was developed with three main functionalities.

The first function is recording the activity sensor data locally as a CSV (comma separated values) file for later training purposes. For each subject, the data were saved on a phone with a unique name. As it could be tiresome to separate different activity data, the recording was carried out in a manner such that each subject recorded one activity at a time. This makes the labeling effort easier. Eight daily physical activities, including walking, jogging, jumping, walking upstairs, walking downstairs, still (standing and sitting), lying down, and bicycle riding were chosen for this research. By taking into account the speed or rhythm that humans perform these activities, 50 Hz was chosen as the rate at which sensor readings were recorded. Unlike most HAR works, an outdoor environment was chosen and subjects were asked to start and stop the recording without being told how to perform the activities. A total of eight subjects participated in the data collection process for approximately 19 min each. An aggregate of approximately 150 min of activity data were gathered for deep learning training.

The second function of the iOS application is to stream real-time sensor data to a computer during real-time inferencing. During testing the trained models on the computer, the smartphone transmits real-time activity data via Wi-Fi to the computer. Hence, both terminals must be connected to the same wireless router. The rate at which the smartphone communicates with the computer was at the same rate as the data were collected, which was 50 Hz. This is how the efficacy of the trained neural network models was validated using an actual device.

Finally, the third task of the application is real-time activity recognition on the smartphone itself. Real-time activity inferencing/prediction was investigated not only on a computer but also on a smartphone by embedding the model into the application software. This can be seen in the above Figure 5d.

### 3.2. Data Preparation

#### 3.2.1. Data Segmentation

**Figure 6:**
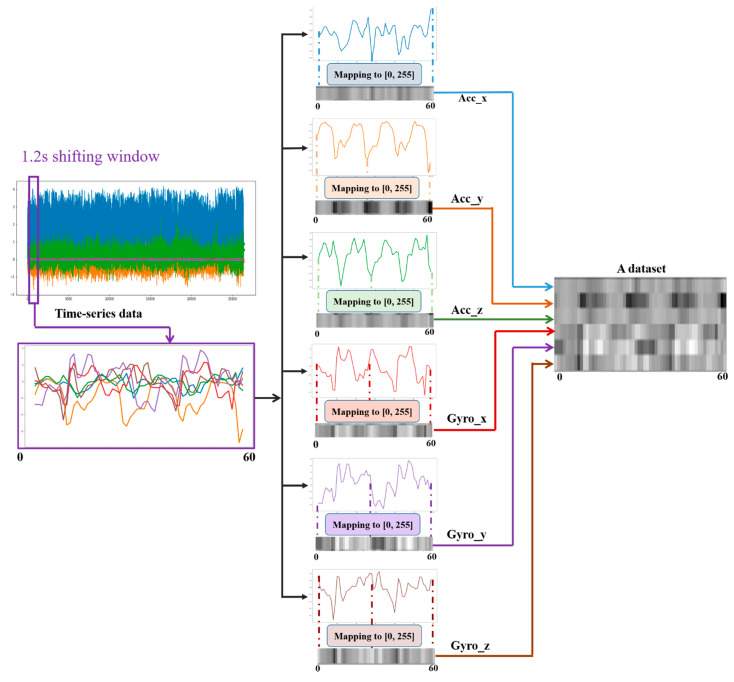
Mapping a time series data segment into a grayscale image.

As many human activities are a repetition of a single motion (e.g., walking is the repetition of steps), the large time series data were divided into small data frames by using a sliding window of a certain time interval. In [83], the cadence of a normal walking person was observed to be 90 steps/min (0.67 s per step). Considering the frequency of repetition of all the activities, a 1.2-s sampling window size with 50% overlap was considered so that activities with a slower period could benefit from a wider range, as shown in Figure 6. A sampling window size that is too large and too small has a significant effect on the overall performance of the system. If the size is too large, a single sampled frame could include multiple activities, which will be wrongly considered as a single activity. On the contrary, a small window could result in insufficient data for makeup even for a single activity. As described above, 50 Hz, which indicates 50 sensor readings per second, was set for the data collection stage. Hence, a 1.2-s shifting/sampling window represents 1.2 × 50 = 60 sensor readings per dataset. This is how datasets are formed for neural network training. Having a 50% overlap in the shifting window helps us double the quantity of the training dataset. As shown in Figure 6, an individual dataset is depicted in grayscale image form by mapping the maximum and minimum values to 0 and 255. A particular Python module was used to perform this.

**Figure 7:**
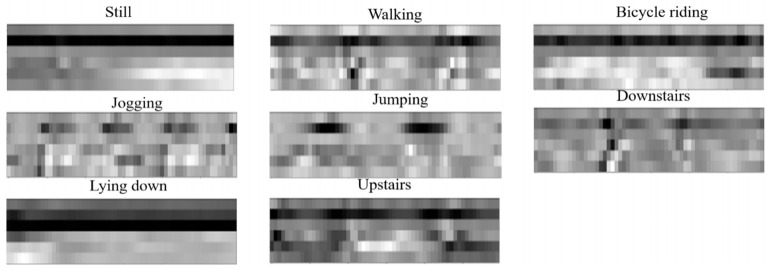
Examples of datasets in grayscale format.

To increase the diversity of the datasets and improve the generalization of deep learning methods, a previous work by [84] proposed the application of translation, rotation and noise injection during the training and testing of natural images. However, in the case of virtual images, as shown below in Figure 7, such augmentations could result in a meaningless dataset as the sensor is fixed in position and orientation, unlike cameras, which enable us to take pictures from different angles. For example, if we flip a walking dataset horizontally, it will have a physical meaning of walking backwards which is not a physical activity. Furthermore, adding more pre-processing would increase the computational cost during the real-time HAR. Hence, a single augmentation, which is a 50% overlapping of the shifting window is used to create the raw datasets.

#### 3.2.2. Data Representation

To investigate a dataset representation exhibiting superior activity characteristics during classification, time series data were represented in different formats by transforming the raw data. Three of the most common time series data representations are appraised here; these are time-domain (TD), frequency-domain (FD), and wavelet transformation (WT). Each type of representation is discussed in detail below.

The TD representation is the raw time series sensor data collected from the smartphone application software by itself, as shown in Figure 6 and Figure 7. For an FD representation, a Fourier transformation is performed on the given time series. FFT (fast Fourier transformation) determines the DFT (discrete Fourier transformation) of an input signal significantly faster than directly computing it by minimizing the number of operations required for a problem. The frequency domain is particularly vital for breaking down a signal comprising of multiple pure frequencies. From a frequency perspective, daily activities are repetitive actions. For instance, walking and jogging both include the cycle of the landing and swinging of the lower limbs. However, the frequency of these cycles is different in both activities, wherein it is faster while jogging than while walking. In addition, the magnitude of the frequency signals from jogging was higher than that of walking, owing to the higher speed and acceleration that could be captured by the IMU sensor. Thus, when it comes to daily activities, picking signals with pure frequency values is easier. However, these activities vary according to their frequencies and magnitude. Therefore, FD representation (amplitude vs. frequency) is a competitive candidate for a good representation of time series signals.

**Figure 8:**
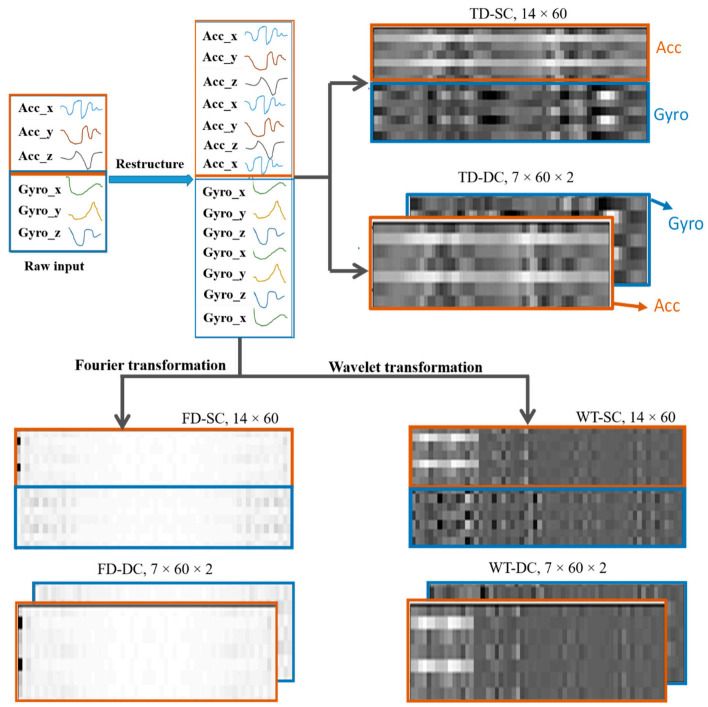
Virtual images of input data representations. TD-SC (time-domain-single channel), TD-DC (time-domain-double channel), FD-SC (frequency domain-single channel), FD-DC (frequency domain-double channel), WT-SC (wavelet transformation-single channel), WT-DC (wavelet transformation-double channel).

The white part of the FD grayscale images in Figure 8 represents higher-amplitude signals. The magnitude of the amplitude decreased with the brightness of the pixels. Hence, the figure shows that the stronger signals have a medium frequency, as can be seen from the white part of the figure. DC components, which have a very small frequency, on the left side and high-frequency sensor noises on the right part of the figure can be easily observed.

From FFT representation, we obtain a frequency spectrum of the real signal but we do not know when that “frequency” occurs. The time resolution of the signal is lost. To obtain both frequency and time resolution, we can divide the original signal into several parts and apply FFT to each part. This is known as the WT. WT is computed for different segments of the signal by translating and dilatation factors. While the FFT has a uniform time–frequency distribution, the wavelet transform provides a multi-resolution analysis.

As can be seen from the FD images in Figure 8, the most important frequencies of the activities occur around the center of the frequency range. Three-level decomposition discrete WT, which results in four coefficients (three approximation coefficients and one detail coefficient), can be accurately able to represent the signals. Hence, a three-level decomposition was performed on the sensor data. The wavelet coefficients obtained are shown on the right side of the lower figure of Figure 8.

#### 3.2.3. Data Structuring

The structure of the input data has a significant influence on the deep learning performance results. To obtain a better input adaptation, three different input data structures were studied here. These are unaltered, single-channel and double-channel structured versions. As described previously, six IMU sensors (three accelerometers and three gyroscopes) were utilized for data collection.

As each dataset has a length of 60 sensor readings, there are a total of six signals of a length of 60 readings each. If we arrange them vertically, a 6 × 60 array of data is acquired. This is denoted as the unaltered version of the input data, where the raw signals are shaped into a simple array that can be regarded as a virtual image. As the name indicates, a virtual image does not represent any object’s drawing, but data are shaped in the same manner as images are represented. As a result, 6 × 60 virtual images were formed, where the upper and lower 3 × 60 data frames were accelerometers and gyroscope readings, respectively.

To extract more deep features, not only from the temporal axis but also from the spatial correlation of the sensor signals, each sensor’s data frame (3 × 60) is restructured into a 2D virtual image of shape 7 × 60. This is produced by duplicating the signals once more to obtain uniformly distributed signal interdependencies when it is applied for neural network training. Duplicating it more than twice would add unwanted redundancy. On the contrary, leaving it unaltered would result in an untimely disappearance of features before being exposed to the network. Hence, to avoid these issues, a new restructuring is introduced, as shown in Figure 8. When both virtual images of the two sensors are piled on top of one another to create a 14 × 60 activity virtual image, which is denoted as SC (single-channel).

For time series signals, the correlation among signals is one of the vital features that need to be extracted for effective neural network classification. Hence, the raw sensor data are restructured, as shown in Figure 8. The data of both sensors are arranged in the x, y, z, x, y, z, x order. The correlations can be between two signals (e.g., x-y, y-z) and among all three (e.g., x-y-z). If we assume only the correlation of two spatial signals of one of the sensors, the output will be xy, yz, zx, xy, yz, and zx. Three duplicate correlations were obtained. The duplication will help us in that important features of the input signal’s traits will not vanish early in the network before being extracted, especially when using the CNN, where pooling layers are employed to significantly reduce the data dimension. Furthermore, features are not produced solely from the correlation of two signals; hence, the duplication will help the network to extract other deeper features in the later neural network layers by keeping the signals’ information alive uniformly throughout the network.

Finally, the 7 × 60 data frames of both the accelerometer and gyroscope are stacked side by side to form a two-channel virtual image, denoted as DC (double-channel). The channel is introduced to avoid mixed-up convolution of different sensor measurements (accelerometer and gyroscope) in the same kernel of an early stage of the network. Hence, different types of data on different channels can be learned using independent kernels. Generally, data structuring considers spatial and temporal correlations, computational time, learning sensor-specific features separately, and on-device applications into consideration.

**Figure 9:**
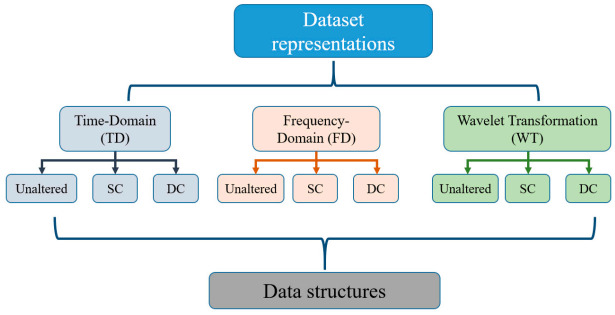
Input data representation and structuring.

Finally, considering the three data representations structured in three forms of virtual images in Figure 8, the input data adaptation structure is summarized, as shown below in Figure 9.

## 4. Neural Network Architecture

Modeled to mimic the human brain operation, a neural network comprises thousands or even millions of nodes that are densely interconnected with each other. Usually, neural networks are arranged into layers of nodes, and they are “feed-forward,” meaning that data move through them from the input side to the output side in only one direction. As our brain utilizes neuron firing to perform various tasks and processes, neural networks use an activation function, that decides whether it should fire (activate) a node.

There are several activation functions, that perform well with different deep learning problems. Among these functions, the most popular are step, linear, sigmoid, tanh, ReLU (rectified linear unit), and softmax. These are their associated pros and cons. In particular, ReLU can work effectively in multiclass classification with a lower probability of gradient vanishing. In addition, as all negative inputs are mapped to zero, the network can have sparse activation. Imagine a network with randomly initialized weights and almost 50% of the network yields zero activations owing to the ReLU characteristics. This indicates that fewer neurons are firing (sparse activation), and the network is lighter. Usually, the number of nodes is in the hundreds of thousands if not in millions. Hence, employing ReLU, which is a computationally less expensive mathematical operation, has a significant effect on the computation time of the entire network. Softmax was also used on the last layer of the neural networks to predict the likelihood of predicting a class.

During training, methods such as exponentially decreasing the learning rate and batch normalization were used to speed up the training process. Selecting the right learning rate is difficult because a too-small value may result in a long training process that could get stuck, whereas a considerably large value may result in learning a sub-optimal set of weights too fast or swings around the optimal solution which is an unstable training process.

In the first steps of the training process, the weight values are most likely too far from the optimum values. Hence, the learning rate should be relatively large at first and as the training progresses, we want the learning rate to be smaller so that we can jump over the best possible solution. The learning rate initially set to 0.0001 was exponentially decayed at a rate of 0.9, with a step size of 1000.

Batch normalization is a technique for training very deep neural networks by standardizing the inputs to a layer for each mini-batch dataset. It reduces the effect of the internal covariance shift of activations, which forces each mini-batch input of a layer to have similar distribution throughout the hidden layers, as described in the original paper [22]. This stabilizes the learning process and reduces the number of training epochs required to train deep networks.

All parameters of the neural network models were updated through the Adam optimizer using the back-propagation principle [85]. A mini-batch size of 50 was selected to train the networks for approximately 100 epochs. The exponentially decreasing learning rate parameters, mini-batch size, LSTM units, and other hyperparameters were determined after several training trials. As a result, the trial-and-error method helped us to find out model parameters that result better and smoother results.

### 4.1. Convolutional Neural Network

A CNN can successfully capture the spatial and temporal dependencies in an image through the application of relevant filters. It is composed of convolution layers, pooling layers, and fully connected layers. The convolution layers are the core part of the CNN, where the dot product of weights (kernels) and input data followed by a ReLU function are performed. The kernels are usually smaller in size than the input data and must move from left to right, starting from the top part down to the bottom depending on the shifting parameter known as stride that decides the number of steps the kernel has to move between convolutions. The activation function then performs an element-wise operation on each input of the corresponding layer.

The other part of the CNN model is a pooling layer that follows the convolution layers. The pooling layer is responsible for reducing the spatial size of the convolved feature. This decreases the computational power required to process the data through dimensionality reduction. Furthermore, it is useful for extracting dominant features that are rotational and positional invariant, thus maintaining the process of effectively training the model. There are two types of pooling methods: max pooling and average pooling, where the former takes the maximum value, and the latter performs an average over the pooling window. As there are not many extreme features in most daily activities’ datasets, average pooling was chosen.

Finally, an FC (fully connected) and an eight-class softmax classifier are added at the end of the network. The FC is where the multidimensional array of data is flattened and provided to the classifier.

**Figure 10:**
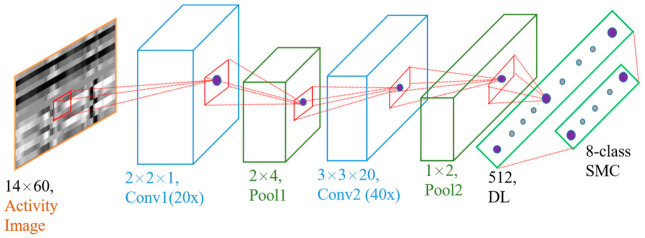
SC-CNN model. Conv = convolution layer, pool = pooling layer, DL = dense layer (fully connected), and SMC = softmax classifier.

The softmax function is a function that transforms a vector of “k” real values into a vector of “n” real values whose sum is 1. The input values can be positive, negative, zero, or greater than one, but the softmax transforms them into values between 0 and 1 so that they can be interpreted as probabilities. The class with the highest probability value was deemed to be the predicted class of the input data. The entire CNN model is shown in Figure 10.

Owing to the three types of data structuring, two CNN models were prepared: single-channel CNN (SC-CNN) and double-channel CNN (DC-CNN). The former is implemented over the unaltered and single-channel structures of the three data representations. The latter is performed over double-channel versions of the data structures. For the SC-CNN in the first convolutional layer, there are 20 kernels of size 2 × 2 × 1 with a stride length of 1 for all axes, as shown in Figure 10. This configuration allows us to examine the important characteristics of the relation between any two signals of the input activity virtual image data. This produces features from the correlation of x-y, y-z, and z-x repeated twice, forming six rows. It is then followed by an average pooling layer of a 2 × 4 window size. After the pooling, an averaged result of the input data is obtained, which are the averages of x-y and y-z, z-x and x-y, and y-z and z-x. Taking one of the averages, x-y and y-z, it will be mainly influenced by the y-signal’s high features, as y exists on both. The same applies to the other two averages which are mainly influenced by the sharp features of x and z. The second convolution layer takes the output from the first layer and is convolved by another 40 3 × 3 × 20 kernels. The second layer calculates the features based on the interdependence of all combined signals. Again, it is accompanied by a 1 × 2 average pooling layer. Except for the kernel size of the first layer, which is 2 × 2 × 2, the SC-CNN structure also applies to the DC-CNN model.

Each convolutional layer performs a 2D convolution on each of its input channels followed by ReLU, which is a nonlinear activation function. The average pooling method reflects the effect of each element within the pooling window on its output map. The dense layer then vectorizes the result of the second average pooling into a 256-dimensional feature vector in the DC-CNN and 512 in the SC-CNN. Finally, these generalized vectors are fed into an eight-class softmax layer, which determines the activity type based on the probability distribution of the activity classes. The neural network was trained with a mini-batch size of 60 and an exponentially decaying learning rate to speed up the training.

### 4.2. Long Short-Term Memory

Unlike other feedforward neural networks, LSTMs have feedback connections to help them relate past and present information. Hence, they are capable of learning long-term dependencies with the help of memory cells, according to previous works [86]. Unlike other traditional RNNs, LSTMs have four neural network layers, mostly composed of sigmoid and tanh layers interacting in a special fashion. A single LSTM cell has two extra inputs other than the normal input dataset, namely the cell state and cell output from the preceding cell. As a result, it yields two outputs: the cell state and the cell output. An LSTM model can support multiple parallel sequences of input data, such as each time series’ IMU motion data. The network learns to extract features from the sequences of the signals by looking into their temporal dependence. This enables them to learn directly from raw time series data.

**Figure 11:**
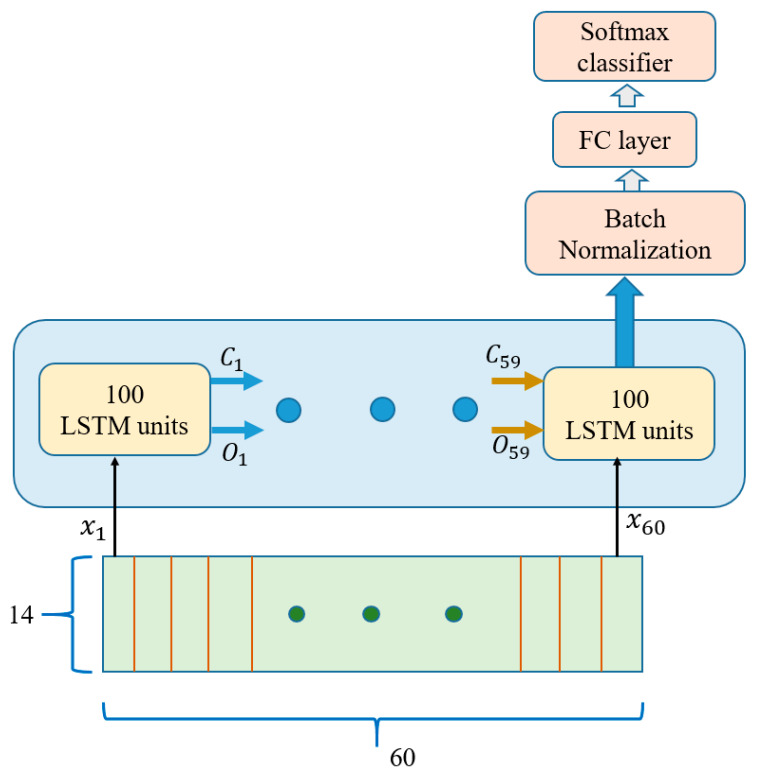
LSTM Network (*x* = input, *C* = cell state, *O* = output, FC = fully connected).

For their good record in sequential data learning, LSTMs are relevant network models for IMU sensor datasets training. As mentioned in Section 3, our datasets have a length of 60 sensor readings on the temporal axis. Hence, each dataset was divided into 60 time steps for LSTM network training. Each IMU sensor value in each dataset was fed to 100 hidden units of the LSTM cells. These cells were accompanied by a dense layer and an eight-class softmax that output the appropriate activity class corresponding to the input. The network used is shown below in Figure 11.

### 4.3. ConvLSTM

ConvLSTM was developed by [75] for predicting precipitation intensity nowcasting. It is a type of recurrent neural network and is used especially for spatiotemporal prediction, which has convolutional structures in both the input-to-state and state-to-state transitions. It is very similar to LSTM units, except in that the internal element-wise operations are replaced by convolution operations. As a result, the input data retains its dimensions while it passes through the LSTM network units. In our case, a 1 × 3, 64 convolution kernels were used to learn the dataset features.

## 5. Experimental Results and Discussion

The deep-learning algorithms were programmed and trained on a computer with 16GB RAM, 3.6-GHz Core-i7 embedded processor, and an NVIDIA GeForce GTX type GPU (graphical processing unit) using Python 3.6. A summary of the experimentation setup and devices used is shown below in Table 1. For the case machine learning libraries, there are several open sources that provide lower and higher APIs (application programming interfaces) to deep learning methods. For its user-friendliness and simplicity, a high-level API named Keras is used for training and inference. Keras is a high-level API of TensorFlow: an approachable, highly productive interface for solving machine learning problems, with a focus on modern deep learning. It provides essential abstractions and building blocks for developing and shipping machine learning solutions with a high iteration velocity. Keras empowers users to take full advantage of the scalability and cross-platform capabilities of TensorFlow running on CPUs (central processing units), TPUs (tensor processing units), and on clusters of GPUs. The trained models can be saved to run them later on a browser or on a mobile device [87].

In our dataset, eight male subjects participated in the data collection process for the eight activities. The subjects had an age range of 25 ± 4 years and were from Asia, Africa, and America. The total number of datasets recorded was 14,962, which were split into 11,962 (79.9%) training datasets, 2000 (13.4%) validation sets, and the remaining 1000 (6.7%) development sets. The entire dataset is composed of 19.8% walking, 9% jumping, 13% upstairs, 12.8% downstairs, 10.4% jogging, 14.5% bike riding, 11.5% still, and 9% lying down data. The training datasets were the main part of the datasets used to train the neural network. In addition, every time the model is updated during its training phase, the newly updated model is tested on the validation datasets. The effectiveness of a model can be investigated based on its performance on the validation datasets. Often, a significant gap between the training and validation performances can occur. The model can overfit the training data but not the validation data. In contrast, it can perform poorly with training data owing to its inability to capture the relation between the input and output data. This is known as an underfitting model. Hence, large, and diversified datasets are required for model training to perform well on both seen and unseen data. Furthermore, the unseen development datasets were used to confirm the generalization capability of the trained model to new input data.

### 5.1. Network Performances

**Figure 12:**
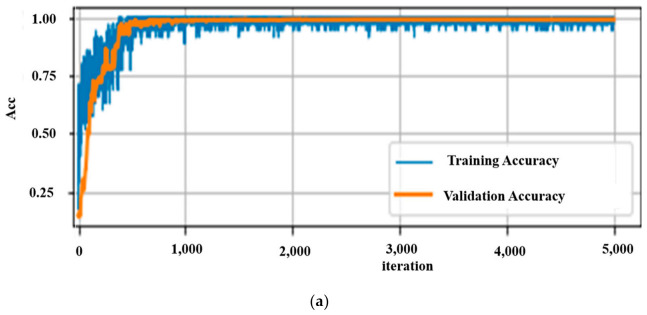

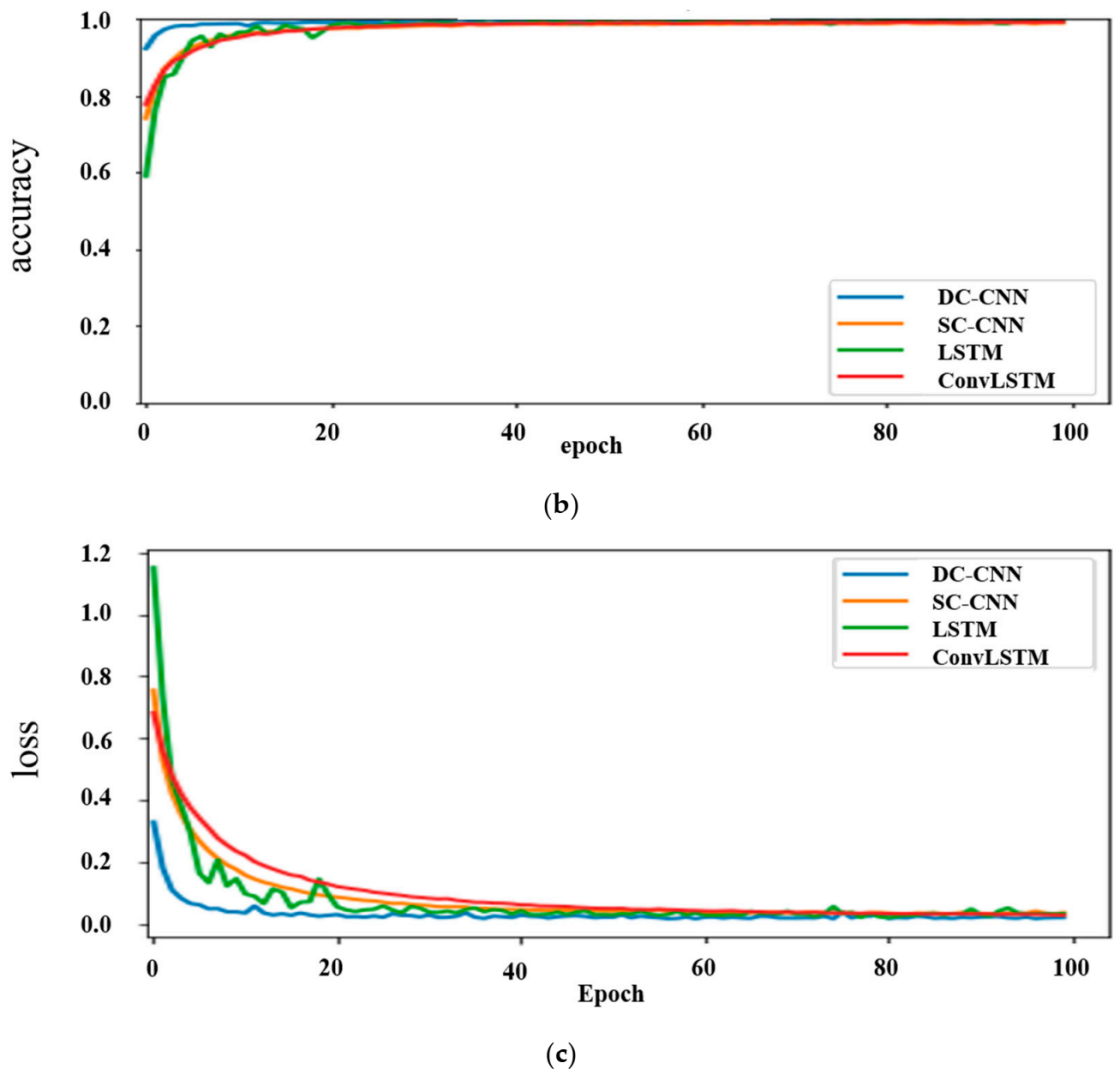
(**a**) DC-CNN accuracies over TD-DC; (**b**) validation accuracies; and (**c**) loss graphs of different models for validation TD dataset.

The performances of the neural network models for the different data structuring methods are presented in Table 2. In addition, the validation accuracies, the time taken per training step, and the number of trainable parameters of each network are shown. For the on-device testing of the models, the computational time and compactness of the model are among the major factors to be considered. Depending on both the time spent for the input data to propagate until the end layer and the performance accuracy of the networks, it is clearly shown that a CNN for a TD-DC performs well. Graphically, the training accuracies, validation/testing performance, and loss of this network together with the others for TD datasets are shown in Figure 12.

The results did not under-fit or over-fit for any of the data representation and neural network combinations since a good result was obtained for both the training and validation datasets. For comparison, only the validation accuracies and respective losses of the different models are shown in Figure 12 (lower two graphs). A validation accuracy of 99.4% with an optimal number of parameters and computational time was achieved. This demonstrates that time series representation with the DC-CNN network can perform quite well in classifying motion data. Hence, this neural network model was chosen as the best model for on-device testing and inferencing. This was tested on both the smartphone and the computer for real-time recognition.

To indicate the improvement obtained from the proposed data structuring, raw data of size 6 × 60 were also used to train the CNN network. As it is a single-channel dataset, SC-CNN was used for training. This result is compared with the DC-CNN result of the TD-DC dataset, as depicted in Table 2. Table 2 shows that, restructuring the raw data into a double-channel form enhanced the accuracy results. The validation accuracy increased from 98.6% to 99.4%.

Finally, the DC-CNN model was evaluated using the development datasets. A confusion matrix is used to display its generalization performance on completely new datasets, as shown in Table 3. Each diagonal of the confusion matrix cell quantifies the number of correctly predicted input datasets of an activity. The other cells indicate the number of incorrectly classified development datasets. For instance, if we take the cell on the third row and column one, it is assigned a value of “3”, which is interpreted as three upstairs ascending datasets that were wrongly predicted as a walking activity.

### 5.2. Public Datasets

Even though an excellent result was obtained from the datasets collected in this research, it is always good to re-evaluate the methodologies discussed so far with other related previous works. There are a couple of HAR works on the Internet, but those with open access public datasets were chosen to investigate the methods developed in this research. Four popular public datasets were selected to evaluate the DC-CNN model and TD-DC data structuring methods. Similarly, public human activity datasets were collected from a smartphone. The datasets are the UCI dataset as found in [11], the WISDM public dataset from the work of [23], the physical activity recognition dataset [12], and UniMib SHAR [89].

#### 5.2.1. WISDM Dataset

This was collected and organized by Fordham University’s Wireless Sensor Data Mining Laboratory. However, unlike the other public datasets, this dataset contains only the accelerometer data of a smartphone connected to the waist. They used a 20 Hz sampling rate to record the motion data. Hence, when a 1.2 s shifting window was used to downsize the data into dataset pieces, a single dataset had a length of 24 accelerometer readings. In addition, a 50% overlap of the sampling window was applied when dividing the dataset into small frames. The fact that this dataset has only accelerometer sensor values forces us to structure it into a single-channel of 7 × 60 virtual image form. Based on this, an SC-CNN model with TD-SC data structuring was utilized for classification. In [23], a J48 decision tree algorithm was used for classifying the WISDM dataset, as shown in Table 4. The same dataset was used to fit the SC-CNN model and the result is also shown in Table 4, where our method is significantly better in the classification of the activities.

#### 5.2.2. UCI Dataset

This work was conducted at the University of California’s Center for Machine Learning and Intelligent Systems. It was used in [11] for wearable sensor-based HAR work. Fourier transform was carried out over the raw data and restructured into a 36 × 68 dimension array to fit a D-CNN (deep-convolutional neural network). Ref. [20] also studied the 1D CNN implementation over a six-input channel (three-accelerometer and three-gyroscope). The sampling rate for the data collection of the UCI dataset was 50 Hz, which is the same as this study. Hence, a TD-DC structuring was used to train the DC-CNN to compare the results, as shown in Table 4. However, our study shows its superiority over the original work.

#### 5.2.3. Physical Activity Recognition Dataset

This is one of the recent datasets released and it includes data for eight daily physical activities with seven body-worn sensors. Among the sensors, only the chest sensor’s accelerometer and gyroscope data were captured to make an equivalence with what this paper is using as motion input data. Fifteen subjects, seven females and eight males with an age of 31.9 ± 12.4 years, a height of 173.1 ± 6.9 cm, and a weight of 74.1 ± 13.8 kg participated in this dataset. Each subject performed each activity for approximately 10 min, and a total of 1065 min of data were acquired. The random forest learning method was used to classify the manually extracted time and frequency features of the motion data [12].

#### 5.2.4. UniMiB SHAR Dataset

This dataset includes a total of 11,771 accelerometer data samples from 30 subjects performing daily life activities and falling motions. The daily living activity dataset comprised nine types of activities. The classification task of this dataset was originally benchmarked with four different classifiers and two different feature vectors [89]. To compare with these methods, TD-SC structuring accompanied by SC-CNN training was performed on this dataset, as shown in the results in Table 4.

### 5.3. The Application Software

The Core ML and Vision APIs were released by Apple during the iOS 11 announcement. Core ML provides life to machine learning-based applications so that developers can bring machine learning models into their iOS applications. It is optimized for the on-device performance of a broad variety of model types by leveraging Apple hardware and minimizing memory footprint and power consumption [91]. Recently, Apple has launched an on-device training in Core ML, starting from iOS 13. This paves the way to develop intelligent systems on Apple-based products.

**Figure 13:**
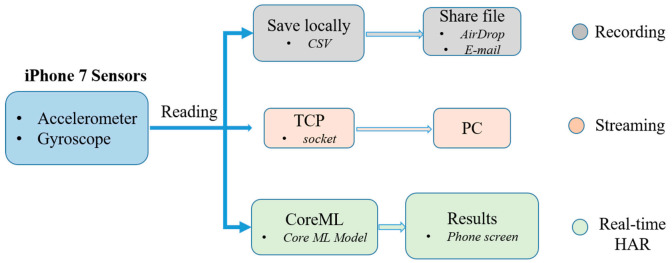
Application program structure.

The smartphone used in this research was an iPhone 7, running iOS 12.1. As discussed in Section 3.1, the application has three functions, as shown below in Figure 13. However, in this section, the third function which is real-time HAR is discussed. To integrate a deep learning model with Core ML, the model should first be converted to a Core ML Model format, which can be manipulated using the Core ML API in the Xcode IDE. Xcode IDE is a platform used in developing applications for Apple products.

**Figure 14:**
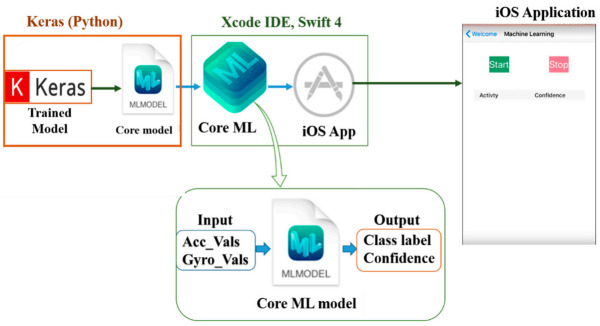
Integration of Keras model and the application.

Depending on the deep learning library, a previously trained model can be converted to a Core ML model using built-in or third-party converters. In our case, Keras’ built-in library, Core ML converter [92], was adopted to convert the Keras model to a Core ML file. The conversion process is illustrated in Figure 14. The generated “*mlmodel*” (an extension of Core ML files) file was then integrated into an iOS application by the Core ML API of the Xcode IDE. After the neural network operation over the continuously fed sensor data was performed, the result was displayed on the screen of the iPhone as a class label and confidence level.

### 5.4. Real-Time Recognition

**Figure 15:**
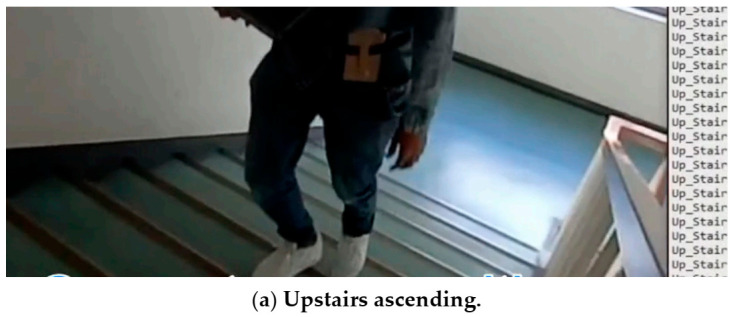

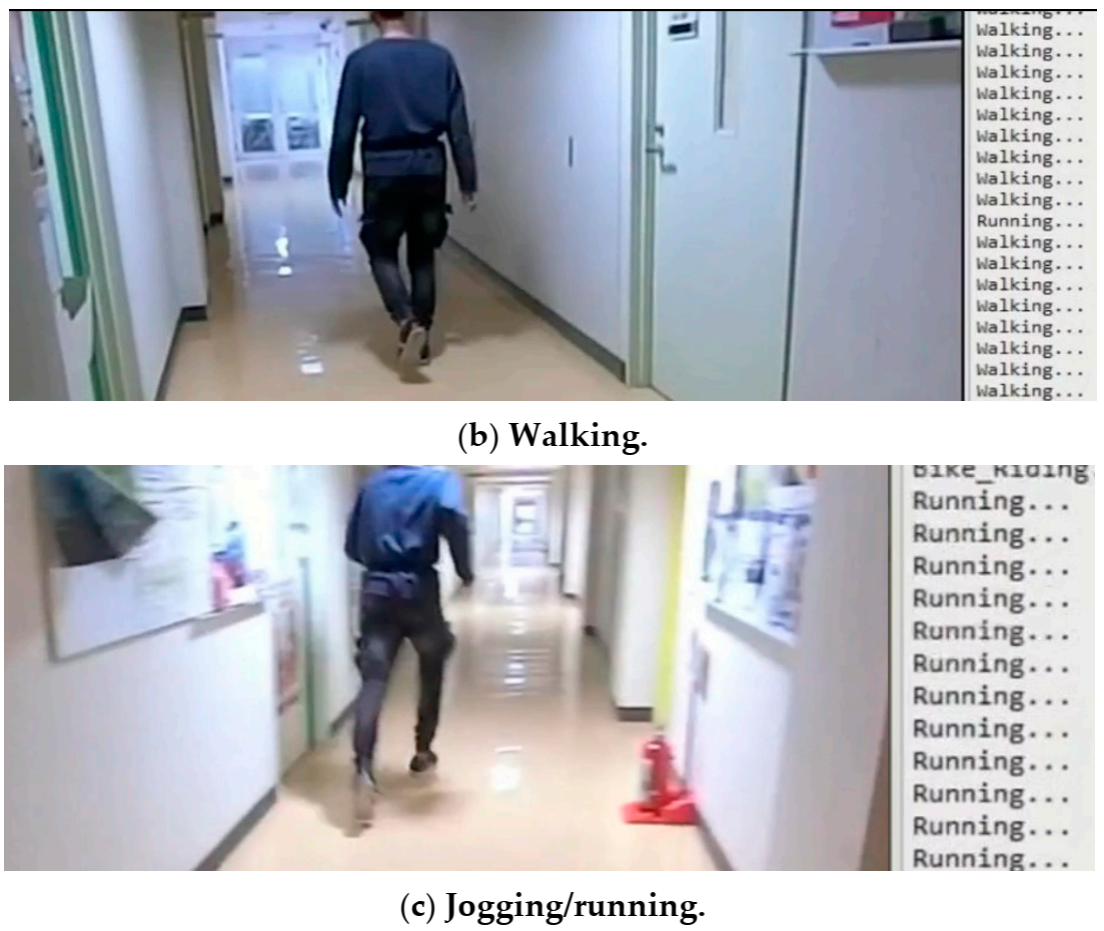
Real-time activity recognition by a computer.

The proposed system was examined in real-time and showed significant results. Before exporting the trained Keras model to the iOS application, it was first tested on a computer for real-time recognition, using the data streaming functionality of the iOS application developed. The phone sends data over a TCP (transmission control protocol) to the computer, where the computer uses a pre-trained model to classify activities in real-time. For the computer, socket programming was used to handle the communication system. In other words, the smartphone was programmed as a server for sending the sensor data while the computer was running a client Python program receiving data at 50 Hz, which is the same rate as the data were first recorded for training. For communication between the devices to be effective, both the smartphone and the computer were connected to the same Wi-Fi or local hotspot connection. Once the connection was established, the received data underwent restructuring to the TD-DC form and neural network operation to output the activity class with its estimated confidence level as shown in Figure 15. Averagely, 80 ms was elapsed for a single dataset to propagate from the data reception to the predicted activity printing.

**Figure 16:**
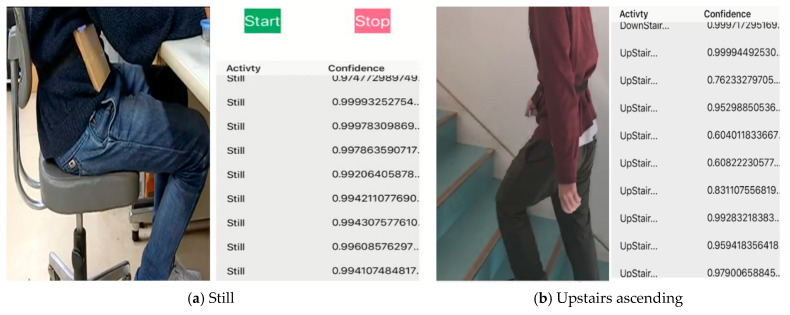

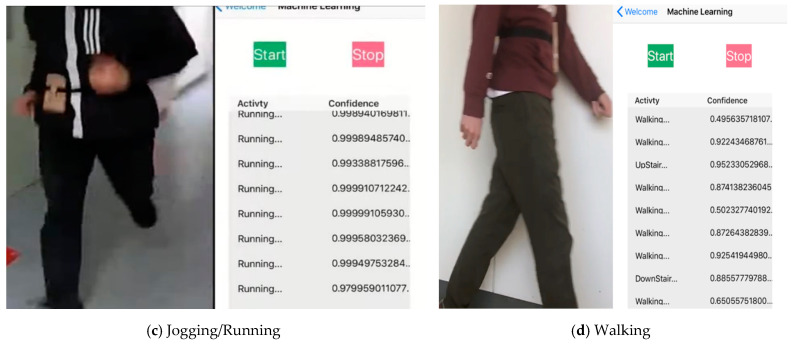
Real-time activity recognition by the iOS application.

As previously discussed, the lighter and effective model, DC-CNN, was incorporated into the iOS program. In the case of using only a smartphone, the phone’s motion data were structured into a TD-DC structured array, using an Xcode library named “MLMultiArray,” and provided as an input to the model. Finally, the type of activity and confidence level are displayed on the screen of the smartphone in tabular form. For both devices, receiving data stream was stopped once previously obtained data started undergoing the inferencing process. The real-time data reception was resumed once the recognition process was completed. This did not introduce too much effect as not a lot of data were ignored in that short time frame. Some snapshots of the iOS application recognition results in real-time are shown in Figure 16.

For new subjects, who did not participate in the data collection, the results were satisfactory for most of the classes. Some of the iOS application recognition results in real-time are shown in Figure 16.

## 6. Conclusions and Future Works

In this study, deep learning of neural networks for HAR was investigated using different devices. A considerable amount of human activity data are required for training. For its omnipresence and convenience, a smartphone was used as a motion sensor to record daily human activities. The smartphone was attached to the waist of the subjects to track the body trunk’s motion. Only the data from the accelerometer and gyroscope sensors were gathered, which were downsized later into 1.2 s dataset partitions as preparation for deep learning. The tiresome labeling process was alleviated using the developed application software for the smartphone.

As neural networks learn by distinguishing the deep features of the input data, various input data structuring methods have been proposed and evaluated to determine the best way to organize input data. In particular, each sensor datum was organized into a 7 × 60 data array formed by duplicating the tri-axial signals. Later, the 7 × 60 data frames from each sensor were combined into single-channel or double-channel virtual 2D image forms. In addition, to the original time series data, two transformations, i.e., Fourier and wavelet transformations were generated on the collected data to increase the data representation.

These different data representations and structures were used to train various neural network models. A structuring method that allows for the extraction of correlation features from each signal independently works well during training. This was confirmed from the validation accuracy of the TD-DC structuring, which was 0.8% higher than the validation accuracy of the unaltered version. In terms of accuracy, computational cost, and training speed, a CNN for the time-domain double-channel (TD-DC) representation input data (DC-CNN), demonstrated better performance than the others. Thus, it remarkably improves the performance of HAR compared to previous state-of-the-art techniques.

To check its effectiveness on other HAR datasets, the methodology was further evaluated using four public HAR datasets. Consequently, it significantly exceeded the related works carried out on public datasets. Furthermore, this model was tested on a computer for real-time HAR and later integrated into a smartphone application. As a result, satisfactory results were achieved for both devices. This demonstrates the practicability of the proposed methodology. Hence, the system can be adapted to different fields where human motion monitoring and analysis are essential, such as safety, healthcare, and human–robot interaction.

Although there has been advances with HAR at the research level, HAR has not made much advancement in real-world applications. Various factors contribute to this phenomenon. However, there is a lack of research focusing on on-device real-time HAR implementation. This, in turn, restricts our knowledge of the challenges and limitations of real-time HAR monitoring. This research will be a basis for further on-device HAR implementations so that we can use HAR on human–robot interaction, society health, and general well-being.

This study had some limitations. The first is that it was performed by attaching a smartphone to the waist of subjects, which is not convenient placement and does not provide comfort to people when adopted for real-world applications. Besides, the proposed system was not applied to data collected from sensors attached to other positions of the human body. Second, this method was not tested on datasets acquired from multiple sensors. After all, there are more than eight human activities, and an increased number of sensors are required to broaden the range of activity recognition. Lastly, the neural network, DC-CNN, is an excellent choice for spatial correlation feature extraction, which could overlook temporal features.

## Figures and Tables

**Table 1:** Devices used for experimentation.

	Devices		
	G-Gear eX.Computer Gaming Series	iMac 27	iPhone 7
Specifications and installed software	64-bit Windows OS system Intel^®^ Core™ i7-7700 CPU @360 GHz 16GB RAM 16GB GeForce GTX 1080 Graphics Keras 2.2.4 installed through Anaconda^®^ Python 3.6	macOS High Sierra Xcode 10.0 Swift 4 CocoaPods 1.6.0 BlueSockets 1.0 [88]	iOS: 12.1 32 GB memory
Purpose	Training and testing	iOS application development	IMU sensor handling and real-time HAR

**Table 2:** Comparison of the models for 100 epochs.

Network	Data Representation	Validation Accuracy (%)	Time/Step (Number of Parameters)
SC-CNN	TD-SC	98.89	240~265 µs (1,176,972 parameters)
	FD-SC	97.90	
	WT-SC	98.46	
	TD-Unaltered	98.60	
	FD-Unaltered	98.30	
	WT-Unaltered	97.80	
DC-CNN	TD-DC	99.40	184.6 µs (621,284 parameters)
	FD-DCWT-DC	98.40 98.56	
LSTM	TD-SC	99.38	2 ms (193,508 parameters)
	FD-SC	97.06	
	WT-SC	97.01	
ConvLSTM	TD-SC	99.20	510 µs (2,560,140 parameters)
	FD-SC	98.83	
	WT-SC	98.86	

**Table 3:** DC-CNN confusion matrix for the development dataset.

True label	Walking	185	0	3	1	0	0	0	0
	Jumping	0	87	0	1	0	0	0	0
	Upstairs	3	0	139	0	0	0	0	0
	Downstairs	0	0	0	116	0	0	0	0
	Jogging	1	0	0	0	109	0	0	0
	Cycling	1	0	0	0	0	141	0	0
	Still	0	0	0	0	0	0	109	0
	Lying	0	0	0	0	0	0	0	104
		Walking	Jumping	Upstairs	Downstairs	Jogging	Cycling	Still	Lying
		Predicted label							

**Table 4:** Comparison of different models over different datasets.

Dataset	Paper by	Data Structure	Network	Accuracy (%)
Own	This paper	TD-DC	DC-CNN	99.40
WISDM [23]	[23]	Heuristic features	J48 decision tree	90.04
	This paper	SC-TD (accelerometer)	SC-CNN	97.08
UCI [11]	[11]	2D FD	Deep-CNN	95.18
	[20]	1D TD	CNN	94.79
	This paper	TD-DC	DC-CNN	97.53
Physical activity recognition dataset [12]	[12]	Heuristic features	Random forest	89.00
	This paper	TD-DC	DC-CNN	94.10
UniMiB [89]	[89]	flattened raw data	kNN	88.51
	[90]	Raw data	Neural network	90.00
	This paper	TD-SC	SC-CNN	95.25

## Data Availability

Code available at: https://github.com/tsgtdss583/HAR (accessed on 1 April 2021).
